# Antimicrobial Resistance Profiles of *Enterococcus faecium* and *Enterococcus faecalis* Isolated from Healthy Dogs and Cats in South Korea

**DOI:** 10.3390/microorganisms11122991

**Published:** 2023-12-15

**Authors:** Bo-Youn Moon, Md. Sekendar Ali, Ji-Hyun Choi, Ye-Eun Heo, Yeon-Hee Lee, Hee-Seung Kang, Tae-Sun Kim, Soon-Seek Yoon, Dong-Chan Moon, Suk-Kyung Lim

**Affiliations:** 1 Bacterial Disease Division, Animal and Plant Quarantine Agency, 177 Hyeksin 8-ro, Gimcheon-si 39660, Republic of Korea; qiamby@korea.kr (B.-Y.M.); alipharm@iiuc.ac.bd (M.S.A.); wlgus01@korea.kr (J.-H.C.); gdd0707@korea.kr (Y.-E.H.); yeonhee6648@naver.com (Y.-H.L.); rkdgmltmd2@gmail.com (H.-S.K.); yoonss24@korea.kr (S.-S.Y.); 2 Public Health and Environment Institute of Gwangju, Gwangju 14502, Republic of Korea; kts2877@korea.kr; 3 Division of Antimicrobial Resistance Research, Centre for Infectious Diseases Research, Korea Disease Control and Prevention Agency, Cheongju 28159, Republic of Korea

**Keywords:** antimicrobial resistance, *E. faecium*, *E. faecalis*, companion animals

## Abstract

*Enterococcus* spp. are typically found in the gastrointestinal tracts of humans and animals. However, they have the potential to produce opportunistic infections that can be transmitted to humans or other animals, along with acquired antibiotic resistance. In this study, we aimed to investigate the antimicrobial resistance profiles of *Enterococcus faecium* and *Enterococcus faecalis* isolates obtained from companion animal dogs and cats in Korea during 2020–2022. The resistance rates in *E. faecalis* towards most of the tested antimicrobials were relatively higher than those in *E. faecium* isolated from dogs and cats. We found relatively higher resistance rates to tetracycline (65.2% vs. 75.2%) and erythromycin (39.5% vs. 49.6%) in *E. faecalis* isolated from cats compared to those from dogs. However, in *E. faecium*, the resistance rates towards tetracycline (35.6% vs. 31.5%) and erythromycin (40.3% vs. 35.2%) were comparatively higher for dog isolates than cats. No or very few *E. faecium* and *E. faecalis* isolates were found to be resistant to daptomycin, florfenicol, tigecycline, and quinupristin/dalfopristin. Multidrug resistance (MDR) was higher in *E. faecalis* recovered from cats (44%) and dogs (33.9%) than in *E. faecium* isolated from cats (24.1%) and dogs (20.5%). Moreover, MDR patterns in *E. faecalis* isolates from dogs (27.2%) and cats (35.2%) were shown to encompass five or more antimicrobials. However, *E. faecium* isolates from dogs (at 13.4%) and cats (at 14.8%) were resistant to five or more antimicrobials. Taken together, the prevalence of antimicrobial-resistant enterococci in companion animals presents a potential public health concern.

## 1. Introduction

*Enterococcus* spp. are Gram-positive opportunistic bacteria generally present in the gastrointestinal tract of humans and animals. They can cause invasive infections in humans, including endocarditis, sepsis, bacteremia, and nosocomial infections of the urinary system and genital tract [1]. Moreover, as commensal bacteria, *Enterococcus* spp. represent a reservoir for host antimicrobial resistance and are considered a good indicator of the effects of selective pressure on antimicrobial use [2]. They can transmit antibiotic resistance to other pathogenic bacterial species through the horizontal transmission of mobile genetic elements [3]. Additionally, the regular use of antibiotics in humans and animals favors the emergence of enterococci resistance, which is difficult to treat [4].

*Enterococcus faecium* and *Enterococcus faecalis* are the most common members, representing more than 90% of the identified species [5]. Moreover, *E. faecium* and *E. faecalis* are known to have high levels of multidrug resistance (MDR), and it has been claimed that they can spread directly from companion animals to humans [6,7]. A previous study found that companion animals treated with antibiotics in an intensive care unit were a source for the zoonotic transmission of MDR *Enterococcus* [8]. The likelihood of the horizontal transmission of zoonotic infections from animals to their owners is increased because companion animals, such as dogs and cats, live in close proximity to their owners. Preventing the spread of MDR *Enterococcus* strains between animals or from animals to humans is crucial for both public health and veterinary medicine. Therefore, research into the characteristics of *Enterococcus* spp. and the potential for the spread of antibiotic resistance is required.

Understanding the antimicrobial resistance profiles of *Enterococcus* spp. isolated from companion animals provides helpful insight into the extent of resistance in the gut microbiota and into antimicrobial selection for treating infections in humans and animals. The antimicrobial resistance profiles of *Enterococcus* spp. isolated from dogs and cats have been described in numerous studies from across the globe [9,10,11,12,13]. The resistance characteristics of *E. faecium* and *E. faecalis* isolates from companion animals have also been studied in various investigations conducted in South Korea [14,15,16,17,18,19]. However, most of these previous studies were conducted to investigate the antimicrobial resistance of *Enterococcus* isolates obtained from diseased animals in a few parts of the country over a very short period of time. Thus, we aimed to conduct this nationwide investigation to determine the antimicrobial resistance profiles and patterns of *E. faecium* and *E. faecalis* isolated from healthy dogs and cats in Korea between 2020 and 2022.

## 2. Materials and Methods

### 2.1. Bacterial Isolates

*Enterococcus* spp. were isolated from feces samples of healthy (with no clinical signs and symptoms of illness) dogs and cats. Sample processing and enterococcal isolation were carried out as described previously [20] using buffered peptone water and *Enterococcus* agar media (Becton, Dickinson, Sparks, MD, USA). Polymerase chain reaction (PCR) and matrix-assisted laser desorption ionization time-of-flight mass spectroscopy (MALDI-TOF, Biomerieux, Marcy-I’Etoile, France) were used to identify *Enterococcus* species [21]. A total of 352 *E. faecium* isolates (298 isolates from dogs and 54 from cats) and 573 *E. faecalis* isolates (448 isolates from dogs and 125 from cats) were collected from eight laboratories/centers that took part in the Korean Veterinary Antimicrobial Resistance Monitoring System (KVARMS) during 2020–2022 (Table S1). In general, the isolates were collected in proportion to the number of different cities in South Korea (Table S2). However, the authors do not have information about the history of antimicrobial use in dogs and cats and the number of samples considered for this study.

### 2.2. Antimicrobial Susceptibility Assessment

We assessed the antimicrobial susceptibility of the enterococcal isolates via the broth microdilution method using commercial antibiotic-containing plates KRVP2 (Sensittitte, Trek Diagnostics, Cleveland, OH, USA). The tested antimicrobials were ampicillin (1–16 µg/mL), chloramphenicol (2–32 µg/mL), ciprofloxacin (0.25–16 µg/mL), daptomycin (0.5–32 µg/mL), erythromycin (1–64 µg/mL), florfenicol (2–32 µg/mL), gentamicin (128–2048 µg/mL), kanamycin (128–2048 µg/mL), linezolid (0.5–16 µg/mL), quinupristin/dalfopristin (1–32 µg/mL), salinomycin (2–32 µg/mL), streptomycin (128–2048 µg/mL), tetracycline (2–128 µg/mL), tigecycline (0.12–2 µg/mL), tylosin (1–64 µg/mL), and vancomycin (2–32 µg/mL). *E. faecalis* ATCC 29212 was used as a quality control strain. The minimum inhibitory concentration (MIC) values were interpreted based on the guidelines of the Clinical and Laboratory Standard Institute [22], the National Antimicrobial Resistance Monitoring System [23], and the Danish Integrated Antimicrobial Resistance Monitoring and Research Program [24]. The lowest concentrations of antimicrobials at which 50% and 90% of the isolates were inhibited were designated as MIC_50_ and MIC_90_, respectively. Multidrug-resistant isolates were defined as those that exhibited resistance to at least three different antimicrobial subclasses [25].

### 2.3. Statistical Analysis

The rates of antimicrobial resistance and Pearson correlation were analyzed using Excel (Microsoft-Excel, 2016, Microsoft Corporation, Redmond, WA, USA) and Rex Software (Version 3.0.3, ResSoft Inc., Seoul, Republic of Korea), in which the independent samples *t*-test, Fisher’s exact test, and chi-square test were incorporated. *p* values < 0.05 were considered statistically significant.

## 3. Results

### 3.1. Antimicrobial Resistance

The resistance rates of *E. faecium* and *E. faecalis* isolates to the tested antimicrobials are displayed in Table 1, Tables S7 and S8. Among them, the highest tetracycline resistance (>65%) was observed in the *E. faecalis* isolates recovered from dogs and cats. Moreover, resistance to tetracycline (34.9 and 67.4%) and erythromycin (39.5 and 41.7%) was high in both *E. faecium* and *E. faecalis*, although the resistance rates differed by bacterial species. Resistance to ciprofloxacin (41.8%) and kanamycin (32.5%) was high in *E. faecium* and *E. faecalis*, respectively. Ampicillin resistance in *E. faecium* was observed in both dogs and cats, with levels >15%, but no ampicillin resistance was observed for *E. faecalis*. Moreover, no or only low resistance to daptomycin, linezolid, tigecycline, and vancomycin was observed in both *E. faecium* and *E. faecalis*.

The overall resistance rates toward the tested antimicrobials in both *E. faecium* and *E. faecalis* isolated from dogs and cats were not significantly different, except for the rate for chloramphenicol. Resistance to chloramphenicol was markedly higher in cats (31.2%) than in dogs (16.7%) (*p* < 0.05). In addition, ciprofloxacin, gentamicin, and kanamycin resistance rates were slightly high in both *E. faecium* and *E. faecalis* in cats. The resistance rates of erythromycin (40.3% vs. 35.2%) and tetracycline (35.6% vs. 31.5%) in *E. faecium* were higher in dog isolates than in cat isolates. It has been observed that *E. faecium* isolates exhibit relatively low resistance to chloramphenicol (0.0–2.3%), daptomycin (1.7–3.7%), and florfenicol (0.0–1.3%). Indeed, compared to cat isolates, dog isolates showed higher rates of chloramphenicol (0.0% vs. 2.3%) and florfenicol (0.0% vs. 1.3%) resistance. The MIC_50_ and MIC_90_ values of the investigated antimicrobials against *E. faecium* isolates obtained from dogs and cats are presented in Supplementary Tables S3 and S4.

The majority of the *E. faecalis* isolates obtained from dogs and cats were highly resistant to tetracycline (67.4%), tylosin (41.4%), erythromycin (41.7%), and kanamycin (32.5%). Although there was no significant difference, erythromycin (39.5% vs. 49.6%), gentamicin (17.9 vs. 28%), kanamycin (31.5 vs. 36%), tetracycline (65.2% vs. 75.2%), and tylosin (39.5% vs. 48%) resistance rates were higher in cat isolates compared to dog isolates. In contrast, no resistance to ampicillin, linezolid, quinupristin/dalfopristin, salinomycin, tigecycline, and vancomycin and very low resistance to ciprofloxacin (3.1% vs. 5.6%) and florfenicol (3.1% vs. 8%) were found in dog and cat isolates. The MIC_50_ and MIC_90_ values of the investigated antimicrobials against *E. faecalis* isolates obtained from dogs and cats are presented in Supplementary Tables S5 and S6.

The antimicrobial resistance among the *E. faecium* and *E. faecalis* isolates from dogs and cats varied with their ages (Table 2 and Table 3). Overall, the resistance rate in *E. faecium* from geriatric dogs (≥11 years) was higher compared with those rates obtained from the puppy and juvenile, mature adult, and senior age groups. It was found that 50% of the *E. faecium* isolates from elderly dogs were resistant to ciprofloxacin. Moreover, the resistance rates toward kanamycin (26.2%), streptomycin (28.6%), and tylosin (26.2%) were significantly higher than those toward other antimicrobials in this age group compared to other age groups. In cats, about 25% of the *E. feacium* isolates were shown to be resistant to the tested antimicrobials. Overall resistance was similar in the kitten and junior as well as adult groups, although resistance to ampicillin and ciprofloxacin was slightly higher in the junior and adult group (Table 2). In *E. faecalis,* there was no difference between the age groups of the dogs. Resistance to erythromycin, tetracycline, and tylosin was high in all age groups. In cats, resistance to ciprofloxacin (11.3% vs. 0–5%), florfenicol (15.1% vs. 0–5.0%), and streptomycin (30.2% vs. 16.7–20.0%) in kittens was higher than in other age groups. Furthermore, resistance to chloramphenicol, gentamicin, kanamycin, and tetracycline was much higher in the senior group, although the number of resistant isolates was lower (Table 3).

### 3.2. Antimicrobial Resistance Patterns

Most *E. faecium* (76.4%) and *E. faecalis* (69.8%) isolates were resistant to at least one of the tested antimicrobials (Table 2 and Table 3). In particular, 36.1% of the *E. faecalis* and 21% of the *E. faecium* isolates exhibited MDR (Table 1). MDR was higher in *E. faecalis* recovered from cats (44%) and dogs (33.9%) than in *E. faecium* isolated from cats (24.1%) and dogs (20.5%). Additionally, *E. faecalis* isolates from dogs (27.2%) and cats (35.2%) were observed to be resistant to five or more antimicrobials. However, *E. faecium* from dogs (13.4%) and cats (14.8%) were resistant to five or more antimicrobials.

In total, there were 72 and 71 MDR combination patterns found in the *E. faecium* and *E. faecalis* isolates, respectively (Tables S9–S12). *E. faecium* typically exhibited ciprofloxacin resistance with tetracycline in the dog isolates (10.4%) and cat isolates (7.4%), whereas erythromycin-resistant *E. faecium* was predominantly found in the dog isolates (20.5%; Table 4). In *E. faecalis* isolated from dogs (22.5%) and cats (24.8%), tetracycline resistance was commonly observed (Table 5). Moreover, tetracycline resistance with chloramphenicol was found in *E. faecalis* isolates obtained from dogs (2.5%) and cats (0.8%). Resistance to six antimicrobials, including macrolides (12.8%), was the most prevalent MDR pattern in the *E. faecalis* specimens isolated from cats (Table 5).

## 4. Discussion

In this investigation, significant proportions of the *E. faecium* and *E. faecalis* isolates recovered from healthy dogs and cats were resistant to one or more antimicrobials. This result could provide a potential strategy for combatting antimicrobial resistance in veterinary and human clinical practice settings.

*Enterococcus* spp. are frequently shown to be resistant to antimicrobials in companion animals worldwide [26,27]. In this study, a considerable portion of enterococci isolates demonstrated resistance to erythromycin, tetracycline, and tylosin. High erythromycin resistance in *E. faecium* and *E. faecalis* isolated from dogs and cats was reported in Korea [15,16,17], China [28], Turkey [29], Belgium [30], Italy [31], and the USA [32], constituting results that concur with our findings. In our study, we found high tetracycline resistance rates in *E. faecium* (34.9%) and *E. faecalis* (67.4%) isolates from dogs and cats. A previous study in Denmark showed tetracycline resistance was high in *E. faecalis* (65%) and *E. faecium* (60%) isolates from dogs [33]. Moreover, consistent with this investigation, high tylosin resistance (41.4%) was described in *E. faecalis* isolated from companion animals in Belgium [26] and the USA [34]. Additionally, the ciprofloxacin resistance (41.8%) in *E. faecium* isolates from dogs and cats was similar to that published in investigations concerning South Korea [18] and Portugal [35].

In this investigation, *E. faecium* specimens isolated from dogs (2%) and cats (7.7%) exhibited relatively low resistance rates to chloramphenicol and gentamicin. However, the chloramphenicol and gentamicin resistance in *E. faecalis* specimens isolated from dogs (16.7 and 17.9%) and cats (31.2 and 28%) was higher but still differed from previously published reports. Jackson et al. [34] reported higher chloramphenicol (85%) and gentamicin (79%) resistance in *E. faecalis* from dog isolates. Nonetheless, the chloramphenicol (5%) and gentamicin (8%) resistance rates were lower in *E. faecalis* specimens from cat isolates reported in the study by De Graef et al. [36]. Moreover, variable levels of chloramphenicol resistance were found in *E. faecium* (17–61%) and *E. faecalis* (9–85%) isolated from dogs and cats in several studies conducted in Asia [16,37] and Europe [38,39]. Despite the fact that chloramphenicol is no longer permitted for use in veterinary practice, the rise of chloramphenicol-resistant *Enterococcus* may be linked to the use of other phenicols, such as florfenicol, or to co-selection with unrelated antimicrobials [40]. Moreover, the recurrent use of gentamicin in companion animals might contribute to the resistance of this antimicrobial [41].

*E. faecium* isolates obtained from dogs and cats exhibited comparatively low resistance to the tested aminoglycosides (kanamycin and streptomycin). However, the resistance rates of these antimicrobials were found to be high in *E. faecalis* isolated from dogs and cats. The *E. faecalis* isolates from dogs (31.5 and 18.8%) and cats (36 and 24%) were resistant to kanamycin and streptomycin, respectively. Highly varied kanamycin (30–87%) and streptomycin (25–95%) resistance rates have been observed in *E. faecium* and *E. faecalis* isolated from companion animals in previous investigations conducted in Korea [17], Portugal [35], Belgium [30], and South Africa [13]. Notwithstanding the fact that enterococci have clinically relevant concentrations, they are the antimicrobials of choice for human enterococcal infections when used with synergic antimicrobials. Consequently, resistance to one or more aminoglycoside antimicrobials might be conferred by aminoglycoside-modifying enzymes acting on various aminoglycoside antimicrobials [42].

Penicillin resistance may limit the range of available treatment options for enterococcal infections [43]. Although no ampicillin resistance was detected in *E. faecalis*, 17.3% of the *E. faecium* specimens isolated from cats and dogs showed resistance to this antimicrobial. This result is consistent with previous findings that *E. faecium* from dog isolates exhibited resistance in the UK (23%) [44] and Egypt (14.3%) [45]. On the contrary, it has been found that *E. faecium* from companion animals in Denmark (76%) [46] and Italy (56.5%) [39] demonstrated relatively high ampicillin resistance. Ampicillin is one of the most effective *β*-lactams against *Enterococcus*, preventing peptidoglycan production [47]. Moreover, the intrinsic tolerance of *β*-lactamase action in *E. faecalis* may be linked to specific penicillin-binding proteins such as pbp5, which has a low affinity for ampicillin [48]. Likewise, it has been demonstrated that ampicillin resistance in *E. faecalis* is conferred by point mutations in the penicillin-binding protein (pbp4) and the overexpression of *β*-lactamase [49].

We observed high erythromycin (39.5 and 41.7%) and tylosin (14.5 and 41.4%) resistance in *E. faecium* and *E. faecalis* isolates from both dogs and cats. Moreover, it was found that there was a gradually increasing tendency toward erythromycin resistance in both of the enterococci strains isolated from dogs and cats throughout the study period. This result is supported by the fact that a significant portion of *E. faecium* and *E. faecalis* isolates from companion animals, particularly dogs and cats, in many countries [7,13,30,39,50] showed resistance to erythromycin (26–96%) and tylosin (21–42%). The increased proportion of isolates resistant to macrolides may also be caused by cross-resistance between erythromycin and tylosin. Additionally, previous studies showed that multidrug-resistant *E. faecalis* from companion animals possesses *erm*(*B*) and *tlrA* genes, suggesting the spread of macrolide resistance genes among the enterococci isolates [39,51].

In this study, the resistance rates toward quinupristin/dalfopristin in *E. faecium* isolates from dogs and cats were 7.4% and 9.3%, respectively. The incidence of quinupristin/dalfopristin resistance in *E. faecium* isolated from dogs was much lower than that previously reported in China (38.04%) [28], Italy (78.4%) [52], and the USA (28.6%) [53] but was consistent with that published in Korea (5%) [16]. Although the administration of quinupristin/dalfopristin to animals is not permitted in Korea, the use of streptogramin and virginiamycin that exhibit quinupristin/dalfopristin cross-resistance may be connected to the development of quinupristin/dalfopristin resistance in *E. faecium* [54]. The incidence of quinupristin/dalfopristin resistance *Enterococcus* in companion animals has the potential to contribute to the occurrence of human infections through frequent and direct interactions.

In our study, we also tested the antibiotic susceptibility of four antimicrobials: daptomycin, linezolid, tigecycline, and vancomycin. These antimicrobials belong to four distinct classes and are not currently approved for veterinary use in Korea [20]. In accordance with previous studies conducted in Korea [17] and other countries [55,56], the prevalence of daptomycin resistance is low among the enterococci isolates obtained from companion animals. The infrequency of daptomycin resistance among enterococci may be attributed to the emergence of resistance to spontaneous mutations in EF0631 genes [57]. Numerous previous studies have documented tigecycline, vancomycin, and salinomycin resistance in *E. faecium* and *E. faecalis* strains obtained from companion animals [52,53,56,58]; however, in our present investigation, all the enterococcal isolates exhibited susceptibility to these antimicrobials. In general, enterococcal isolates demonstrating resistance to clinically significant antimicrobials have the potential to be transmitted directly or indirectly to humans, posing a critical clinical challenge for the treatment of multidrug-resistant infections.

The prevalence of antimicrobial-resistant *E. faecium* and *E. faecalis* isolates among dogs and cats with respect to age variation was assessed to elucidate the emergence of antimicrobial resistance, which could help veterinarians in identifying infections and developing treatment strategies and preventive measures for different age groups of companion animals. In our study, it was observed that most of the antimicrobial resistance in the *E. faecium* isolates from dogs and cats increased with age. The prevalence of resistance in the *E. faecium* isolates obtained from geriatric dogs (aged ≥11 years) was higher than that isolated from the puppy and juvenile, mature adult, and senior age groups. Additionally, the isolates from the mature group of cats (aged 7–10 years) showed significantly higher rates of resistance to erythromycin, gentamicin, and tetracycline than those from other age groups. The resistance rate toward the tested antimicrobials in *E. faecalis* was higher in the puppy and juvenile groups compared to the mature adult, senior, and geriatric groups of dogs. Furthermore, it was found that the rate of resistance to the antimicrobials examined was significantly higher in the senior group (aged ≥11 years) of cats compared to the kitten, junior, and mature groups. Consistent with the findings of a previous study, this investigation demonstrated a high prevalence of antimicrobial resistance in isolates obtained from relatively older companion animals [59]. This propensity for antimicrobial resistance in relatively older companion animals might be due to their exposure to more treatment medications throughout their lives [60]. However, previously published reports still showed mixed antimicrobial resistance dynamics in isolates obtained from animals of varying ages [16].

Most of the *E. faecium* and *E. faecalis* isolates exhibited resistance to at least one antimicrobial, with several resistance patterns observed in both species. The rates of multidrug resistance (MDR) in the *E. faecium* (21%) and *E. faecalis* (36.1%) strains from dogs and cats were found in this study. A previous report showed a similar MDR phenotype observed in *E. faecium* (33.3%) isolated from companion animals in the USA [53]. However, MDR in *E. faecalis* isolates obtained from dogs and cats was reported to be higher in China (52.9%) [55] and Korea (71%) [17]. Moreover, MDR in *E. faecium* (97.9%) was found to be much higher in cats and dogs in a previous study conducted by Ma et al. [55].

In accordance with the findings reported by Miranda et al. [61], it was seen that MDR patterns often encompass the presence of erythromycin, tetracycline, and ciprofloxacin. Moreover, it was found that *E. faecalis* isolates obtained from dogs (43.3%) and cats (81.5%) exhibited resistance to five or more antimicrobials, consistent with previously published reports from Korea [16], Turkey [41], and the USA [53]. The tendency of *Enterococcus* spp. to participate in different types of conjugation may be linked to the presence of MDR, leading to the extensive spread of resistance determinants via plasmids [62]. Moreover, the survivability of MDR enterococci may also trigger their widespread clonal and zoonotic transmission, leaving limited options for treating bacterial infections in humans and animals [63].

## 5. Conclusions

Our study revealed that enterococcal isolates recovered from healthy dogs and cats demonstrated resistance to multiple antimicrobial agents, including those deemed crucial for human health. The prevalence of multidrug-resistant *E. faecium* and *E. faecalis* in companion animals is concerning, particularly considering the limited availability of effective antimicrobials for treating enterococcal infections. This resistance can potentially be transmitted to humans as companion animals share a common habitat with humans and are treated for infections using similar medications. Hence, the judicious selection of antimicrobials and the continuous surveillance of antimicrobial resistance in companion animals may play pivotal roles in mitigating the human health risk associated with *Enterococcus*. In addition, resistance mechanisms and whole-genome sequencing analysis of *E. faecium* and *E. faecalis* isolates also remain to be further conducted to address the risk of transmission from dogs and cats to humans.

## Figures and Tables

**Table 1:** Antimicrobial resistance rates in *Enterococcus faecium* (*n* = 352) and *Enterocoocus faecalis* (*n* = 573) isolated from dogs and cats during 2020–2022 in South Korea.

Antimicrobials	Resistance Rate % (No. of Isolates)							
	*E. faecium*				*E. faecalis*			
	Dogs (*n* = 298)	Cats (*n* = 54)	*p* Value	Subtotal (*n* = 352)	Dogs (*n* = 448)	Cats (*n* = 125)	*p* Value	Subtotal (*n* = 573)
Ampicillin	17.8 (53)	14.8 (8)	0.6301	17.3 (61)	0 (0)	0 (0)	ND	0 (0)
Chloramphenicol	2.3 (7)	0 (0)	0.1552	2.0 (7)	16.7 (75)	31.2 (39)	0.0469	19.9 (114)
Ciprofloxacin	41.3 (123)	44.4 (24)	0.7105	41.8 (147)	3.1 (14)	5.6 (7)	0.5234	3.7 (21)
Daptomycin	1.7 (5)	3.7 (2)	0.47	2.0 (7)	0 (0)	0 (0)	ND	0 (0)
Erythromycin	40.3 (120)	35.2 (19)	0.5296	39.5 (139)	39.5 (177)	49.6 (62)	0.2621	41.7 (239)
Florfenicol	1.3 (4)	0 (0)	0.2929	1.1 (4)	3.1 (14)	8.0 (10)	0.2616	4.2 (24)
Gentamicin	7.4 (22)	9.3 (5)	0.685	7.7 27)	17.9 (80)	28.0 (35)	0.1912	20.1 (115)
Kanamycin	12.1 (36)	16.7 (9)	0.4368	12.8 (45)	31.5 (141)	36.0 (45)	0.602	32.5 (186)
Linezolid	0 (0)	0 (0)	ND	0 (0)	0 (0)	0 (0)	ND	0 (0)
Quinupristin/dalfopristin	7.4 (22)	9.3 (5)	0.685	7.7 (27)	ND	ND	ND	ND
Salinomycin	0 (0)	0 (0)	ND	0 (0)	0 (0)	0 (0)	ND	0 (0)
Streptomycin	15.1 (45)	11.1 (6)	0.4718	14.5 (51)	18.8 (84)	24.0 (30)	0.4837	19.9 (114)
Tetracycline	35.6 (106)	31.5 (17)	0.6097	34.9 (123)	65.2 (292)	75.2 (94)	0.2227	67.4 (386)
Tigecycline	0 (0)	0 (0)	ND	0 (0)	0 (0)	0 (0)	ND	0 (0)
Tylosin	14.4 (43)	14.8 (8)	0.9522	14.5 (51)	39.5 (177)	48.0 (60)	0.3479	41.4 (237)
Vancomycin	0 (0)	0 (0)	ND	0 (0)	0 (0)	0 (0)	ND	0 (0)
MDR	20.5 (61/298)	24.1 (13/54)	0.5999	21.0 (74/352)	33.9 (152/448)	44.0 (55/125)	0.2543	36.1 (207/573)

*p* < 0.05 was considered a significant change in the antimicrobial resistance rate. MDR, multidrug resistance; ND, none-defined.

**Table 2:** Distribution of antimicrobial-resistant *Enterococcus faecium* among different age groups of healthy dogs and cats isolated during 2020–2022 in South Korea.

Antimicrobials	Resistance Rate % (No. of Isolates)								
	Dogs (*n* = 298)					Cats (*n* = 54)			
	1 Year: Puppy and Juvenile (*n* = 66)	2–5 Years: Mature Adult (*n* = 88)	6–10 Years: Senior (*n* = 102)	≥11 Years: Geriatric (*n* = 42)	Subtotal	≤1 Year: Kitten (*n* = 28)	2–6 Years: Junior and Adults (*n* = 22)	7–10 Years: Mature (*n* = 4)	Subtotal
Ampicillin	16.7 (11)	9.1 (8)	22.5 (23)	26.2 (11)	17.8 (53)	10.7 (3)	18.2 (4)	25.0 (1)	14.8 (8)
Chloramphenicol	3.0 (2)	3.4 (3)	1.0 (1)	2.4 (1)	2.3 (7)	0 (0)	0 (0)	0 (0)	0 (0)
Ciprofloxacin	48.5 (32)	35.2 (31)	38.2 (39)	50.0 (21)	41.3 (123)	35.7 (10)	54.5 (12)	50.0 (2)	44.4 (24)
Daptomycin	0 (0)	1.1 (1)	2.9 (3)	2.4 (1)	1.7 (5)	3.6 (1)	4.5 (1)	0 (0)	3.7 (2)
Erythromycin	43.9 (29)	33 (29)	41.2 (42)	47.6 (20)	40.3 (120)	35.7 (10)	31.8 (7)	50.0 (2) ^Δ^	35.2 (19)
Florfenicol	3.0 (2)	1.1 (1)	0 (0)	2.4 (1)	1.3 (4)	0 (0)	0 (0)	0 (0)	0 (0)
Gentamicin	10.6 (7)	2.3 (2)	6.9 (7)	14.3 (6)	7.4 (22)	7.1 (2)	9.1 (2)	25.0 (1) ^Δ^	9.3 (5)
Kanamycin	18.2 (12)	2.3 (2) *	10.8 (11)	26.2 (11) ^#^	12.1 (36)	14.3 (4)	18.2 (4)	25.0 (1)	16.7 (9)
Linezolid	0 (0)	0 (0)	0 (0)	0 (0)	0 (0)	0 (0)	0 (0)	0 (0)	0 (0)
Quinupristin/dalfopristin	7.6 (5)	5.7 (5)	6.9 (7)	11.9 (5)	7.4 (22)	14.3 (4)	0 (0) ^†^	25.0 (1) ^Δ^	9.3 (5)
Salinomycin	0 (0)	0 (0)	0 (0)	0 (0)	0 (0)	0 (0)	0 (0)	0 (0)	0 (0)
Streptomycin	15.2 (10)	8.0 (7)	15.7 (16)	28.6 (12) ^#^	15.1 (45)	10.7 (3)	9.1 (2)	25.0 (1) ^Δ^	11.1 (6)
Tetracycline	36.4 (24)	27.3 (24)	39.2 (40)	42.9 (18)	35.6 (106)	28.6 (8)	31.8 (7)	50.0 (2) ^Δ^	31.5 (17)
Tigecycline	0 (0)	0 (0)	0 (0)	0 (0)	0 (0)	0 (0)	0 (0)	0 (0)	0 (0)
Tylosin	18.2 (12)	9.1 (8)	11.8 (12)	26.2 (11) ^#^	14.4 (43)	14.3 (4)	13.6 (3)	25.0 (1)	14.8 (8)
Vancomycin	0 (0)	0 (0)	0 (0)	0 (0)	0 (0)	0 (0)	0 (0)	0 (0)	0 (0)
MDR	24.2 (16)	13.6 (12)	18.6 (19)	33.3 (14) ^#^	20.5 (61/298)	21.4 (6)	27.3 (6)	25.0 (1)	24.1 (13/54)

* *p* < 0.05 compared with resistance rates in mature adult dogs; ^#^ *p* < 0.05 compared with resistance rates in geriatric dogs; ^†^ *p* < 0.05 compared with resistance rates in junior and adult cats; ^Δ^ *p* < 0.05 compared with resistance rates in mature cats. MDR, multidrug resistance.

**Table 3:** Distribution of antimicrobial-resistant *Enterococcus faecalis* isolates among different age groups of healthy dogs and cats isolated during 2020–2022 in South Korea.

Antimicrobials	Resistance Rate % (No. of Isolates)											
	Dogs (*n* = 448)						Cats (*n* = 125)					
	1 Year: Puppy and Juvenile (*n* = 92)	2–5 Years: Mature Adult (*n* = 148)	6–10 Years: Senior (*n* = 147)	≥11 Years: Geriatric (*n* = 59)	Unkn-own (*n* = 2)	Subtotal	≤1 Year: Kitten (*n* = 53)	2–6 Years: Junior and Adults (*n* = 45)	7–10 Years: Mature (*n* = 20)	≥11 Years: Senior (*n* = 6)	Unkn-own (*n* = 1)	Subtotal
Ampicillin	0 (0)	0 (0)	0 (0)	0 (0)	0 (0)	0 (0)	0 (0)	0 (0)	0 (0)	0 (0)	0 (0)	0 (0)
Chloramphenicol	17.4 (16)	16.2 (24)	17.7 (26)	15.3 (9)	0 (0)	16.7 (75)	32.1 (17)	26.7 (12)	30.0 (6)	66.7 (4) ^†^	0 (0)	31.2 (39)
Ciprofloxacin	4.3 (4)	2.7 (4)	2.0 (3)	5.1 (3)	0 (0)	3.1 (14)	11.3 (6)	0 (0) *	5 (1)	0 (0) ^†^	0 (0)	5.6 (7)
Daptomycin	0 (0)	0 (0)	0 (0)	0 (0)	0 (0)	0 (0)	0 (0)	0 (0)	0 (0)	0 (0)	0 (0)	0 (0)
Erythromycin	41.3 (38)	38.5 (57)	40.1 (59)	39.0 (23)	0 (0)	39.5 (177)	43.4 (23)	55.6 (25)	45.0 (9)	66.7 (4) ^†^	100.0 (1)	49.6 (62)
Florfenicol	4.3 (4)	1.4 (2)	4.1 (6)	3.4 (2)	0 (0)	3.1 (14)	15.1 (8)	2.2 (1)	5.0 (1)	0 (0) ^†^	0 (0)	8.0 (10)
Gentamicin	20.7 (19)	17.6 (26)	15.6 (23)	20.3 (12)	0 (0)	17.9 (80)	32.1 (17)	24.4 (11)	20.0 (4)	50.0 (3) ^†^	0 (0)	28.0 (35)
Kanamycin	38 (35)	31.1 (46)	27.9 (41)	32.2 (19)	0 (0)	31.5 (141)	39.6 (21)	35.6 (16)	20.0 (4) ^#^	66.7 (4) ^†^	0 (0)	36.0 (45)
Linezolid	0 (0)	0 (0)	0 (0)	0 (0)	0 (0)	0 (0)	0 (0)	0 (0)	0 (0)	0 (0)	0 (0)	0 (0)
Quinupristin/dalfopristin	ND	ND	ND	ND	ND	ND	ND	ND	ND	ND	ND	ND
Salinomycin	0 (0)	0 (0)	0 (0)	0 (0)	0 (0)	0 (0)	0 (0)	0 (0)	0 (0)	0 (0)	0 (0)	0 (0)
Streptomycin	19.6 (18)	20.9 (31)	15.6 (23)	20.3 (12)	0 (0)	18.8 (84)	30.2 (16)	20.0 (9)	20.0 (4)	16.7 (1)	0 (0)	24.0 (30)
Tetracycline	68.5 (63)	67.6 (100)	63.3 (93)	59.3 (35)	50.0 (1)	65.2 (292)	66.0 (35)	80.0 (36)	85.0 (17)	100.0 (6) ^†^	0 (0)	75.2 (94)
Tigecycline	0 (0)	0 (0)	0 (0)	0 (0)	0 (0)	0 (0)	0 (0)	0 (0)	0 (0)	0 (0) ^†^	0 (0)	0 (0)
Tylosin	42.4 (39)	37.2 (55)	40.8 (60)	39.0 (23)	0 (0)	39.5 (177)	43.4 (23)	53.3 (24)	45.0 (9)	66.7 (4) ^†^	0 (0)	48.0 (60)
Vancomycin	0 (0)	0 (0)	0 (0)	0 (0)	0 (0)	0 (0)	0 (0)	0 (0)	0 (0)	0 (0)	0 (0)	0 (0)
MDR	38.0 (35)	32.4 (48)	34.0 (50)	32.2 (19)	0 (0)	33.9 (152/448)	45.3 (24)	42.2 (19)	40.0 (8)	66.7 (4) ^†^	0 (0)	44.0 (55/125)

* *p* < 0.05 compared with resistance rates in junior and adult cats; ^#^ *p* < 0.05 compared with resistance rates in mature cats. ^†^ *p* < 0.05 compared with resistance rates in senior cats. MDR, multidrug resistance; ND, none-defined.

**Table 4:** Frequent resistance patterns of *Enterococcus faecium* specimens isolated from healthy dogs and cats during 2020–2022 in South Korea.

No. of Antimicrobials	Dog Isolates (*n* = 298)		Cat Isolates (*n* = 54)	
	No. of Isolates (%)	Most Common Pattern (No. of Isolates, %)	No. of Isolates (%)	Most Common Pattern (No. of Isolates, %)
0	68 (22.8)	–	15 (27.8)	–
1	114 (38.3)	ERY (*n* = 61, 20.5%)	19 (35.2)	CIP (*n* = 7, 13.0%)
2	54 (18.1)	CIP TET (*n* = 31, 10.4%)	7 (13.0)	CIP TET (*n* = 4, 7.4%)
3	13 (4.4)	CIP ERY TET (*n* = 4, 1.3%)	3 (5.6)	AMP STR TET (*n* = 1, 1.9%), CIP ERY SYN (*n* = 1, 1.9%), CIP ERY TET (*n* = 1, 1.9%)
4	9 (3.0)	AMP CIP STR TET (*n* = 3, 1.0%)	2 (3.7)	AMP CIP GEN KAN (*n* = 1, 1.9%), AMP CIP ERY TYL (*n* = 1, 1.9%)
5	7 (2.3)	AMP CIP ERY TET TYL (*n* = 2, 0.7%) CHL CIP ERY TET TYL (*n* = 2, 0.7%) CIP ERY STR TET TYL (*n* = 2, 0.7%)	2 (3.7)	AMP CIP GEN KAN TET (*n* = 1, 1.9%), CIP ERY KAN TET TYL (*n* = 1, 1.9%)
6	8 (2.7)	CIP ERY KAN STR TET TYL (*n* = 3, 1.0%)	3 (5.6)	AMP CIP ERY STR TET TYL (*n* = 1, 1.9%), CIP ERY KAN STR TET TYL (*n* = 1, 1.9%), ERY GEN KAN STR TET TYL (*n* = 1, 1.9%)
7	10 (3.4)	AMP CIP ERY KAN STR TET TYL (*n* = 4, 1.3%)	1 (1.9)	AMP CIP ERY KAN SYN TET TYL (*n* = 1, 1.9%)
8	5 (1.7)	AMP CIP ERY GEN KAN STR TET TYL (*n* = 2, 0.7%)	–	–
9	7 (2.3)	AMP CIP ERY GEN KAN SYN STR TET TYL (*n* = 4, 1.3%)	2 (3.7)	AMP CIP ERY GEN KAN SYN STR TET TYL (*n* = 2, 3.7%)
10	2 (0.7)	AMP CIP DAP ERY GEN KAN SYN STR TET TYL (*n* = 2, 0.7%)	–	–
11	1 (0.3)	AMP CHL CIP ERY FLR GEN KAN SYN STR TET TYL (*n* = 1, 0.3%)	–	–

AMP, ampicillin; CIP, ciprofloxacin; CHL, chloramphenicol; DAP, daptomycin; ERY, erythromycin; FLR, florfenicol; GEN, gentamicin; KAN, kanamycin; SYN, quinupristin/dalfopristin; STR, streptomycin; TET, tetracycline; TYL, tylosin.

**Table 5:** Frequent resistance patterns of *Enterococcus faecalis* isolated from healthy dogs and cats during 2020–2022 in South Korea.

No. of Antimicrobials	Dog Isolates (*n* = 448)		Cat Isolates (*n* = 125)	
	No. of Isolates (%)	Most Common Pattern (No. of Isolates, %)	No. of Isolates (%)	Most Common Pattern (No. of Isolates, %)
0	147 (32.8)	–	26 (20.8)	–
1	101 (22.5)	TET (*n* = 101, 22.5%)	33 (26.4)	TET (*n* = 31, 24.8%)
2	22 (4.9)	CHL TET (*n* = 11, 2.5%)	3 (2.4)	CHL TET (*n* = 1, 0.8%), CIP TET (*n* = 1, 0.8%), ERY TET (*n* = 1, 0.8%)
3	25 (5.6)	ERY TET TYL (*n* = 20, 4.5%)	8 (6.4)	ERY TET TYL (*n* = 6, 4.8%)
4	31 (6.9)	ERY KAN TET TYL (*n* = 11, 2.5%)	11 (8.8)	CHL ERY TET TYL (*n* = 8, 6.4%)
5	56 (12.5)	ERY KAN STR TET TYL (*n* = 27, 6.0%)	10 (8.0)	ERY GEN KAN TET TYL (*n* = 3, 2.4%), ERY KAN STR TET TYL (*n* = 3, 2.4%)
6	40 (8.9)	ERY GEN KAN STR TET TYL (*n* = 18, 4.0%)	21 (16.8)	CHL ERY GEN KAN TET TYL (*n* = 8, 6.4%) ERY GEN KAN STR TET TYL (*n* = 8, 6.4%
7	20 (4.5)	CHL ERY GEN KAN STR TET TYL (*n* = 16, 3.6%)	6 (4.8)	CHL ERY GEN KAN STR TET TYL (*n* = 6, 4.8%)
8	4 (0.9)	CHL CIP ERY FLR GEN KAN TET TYL (*n* = 3, 0.7%)	6 (4.8)	CHL ERY FLR GEN KAN STR TET TYL (*n* = 4, 3.2%)
9	2 (0.4)	CHL CIP ERY FLR GEN KAN STR TET TYL (*n* = 2, 0.4%)	1 (0.8)	CHL CIP ERY FLR GEN KAN STR TET TYL (*n* = 1, 0.8%)

CIP, ciprofloxacin; CHL, chloramphenicol; ERY, erythromycin; FLR, florfenicol; GEN, gentamicin; KAN, kanamycin; STR, streptomycin; TET, tetracycline; TYL, tylosin.

## Data Availability

All data generated for this study are contained within the article/Supplementary Materials.

## Supplementary Materials

The following supporting information can be downloaded at: https://www.mdpi.com/article/10.3390/microorganisms11122991/s1, Table S1: *Enterococcus faecium* and *Enterococcus faecalis* isolates recovered from healthy dogs and cats during 2020–2022 in South Korea; Table S2: *Enterococcus faecium* and *Enterococcus faecalis* isolate collection cities in South Korea; Table S3: The MIC_50_ and MIC_90_ of the tested antimicrobials against *E. faecium* (*n* = 298) isolated from healthy dogs during 2020–2022 in South Korea; Table S4: The MIC_50_ and MIC_90_ of the tested antimicrobials against *E. faecium* (*n* = 54) isolated from healthy cats during 2020–2022 in South Korea; Table S5: The MIC_50_ and MIC_90_ of the tested antimicrobials against *E. faecalis* (*n* = 448) isolated from healthy dogs during 2020–2022 in South Korea; Table S6: The MIC_50_ and MIC_90_ of the tested antimicrobials against *E. faecalis* (*n* =125) isolated from healthy cats during 2020–2022 in South Korea; Table S7: Antimicrobial resistance rate in *Enterococcus faecium* isolated from healthy dogs (*n* = 298) and cats (*n* = 54) during 2020–2022 in South Korea; Table S8: Antimicrobial resistance rate in *Enterococcus faecalis* isolated from healthy dogs (*n* = 448) and cats (*n* = 125) during 2020–2022 in South Korea; Table S9: Antimicrobial resistance patterns of *Enterococccus faecium* isolated from healthy dogs (*n* = 298) during 2020–2022 in South Korea; Table S10: Antimicrobial resistance patterns of *Enterococccus faecium* isolated from healthy cats (*n* = 54) during 2020–2022 in South Korea; Table S11: Antimicrobial resistance patterns of *Enterococccus faecalis* isolated from healthy dogs (*n* = 448) during 2020–2022 in South Korea; Table S12: Antimicrobial resistance patterns of *Enterococccus faecalis* isolated from healthy cats (*n* = 125) during 2020–2022 in South Korea.
