# Supplementary material for: Antimicrobial Resistance Profiles of Enterococcus faecium and Enterococcus faecalis Isolated from Healthy Dogs and Cats in South Korea
Source: Microorganisms. 2023 Dec 15;11(12):2991. doi: 10.3390/microorganisms11122991 (PMC10745814; doi:10.3390/microorganisms11122991)
Supplement: Supplementary file 1 [file microorganisms-11-02991-s001.zip › microorganisms-2764392-supplementary.pdf]

Table S1. *Enterococcus faecium* and *Enterococcus faecalis* isolates recovered from healthy dogs and cats during 2020–2022 in South Korea.

Year	*E. faecium*				*E. faecalis*			
	Dogs		Cats		Dogs		Cats	
	No. of animal hospitals	No. of isolates	No. of animal hospitals	No. of isolates	No. of animal hospitals	No. of isolates	No. of animal hospitals	No. of isolates
2020	20	83	11	18	27	175	13	41
2021	21	77	10	14	26	124	14	39
2022	24	138	9	22	25	149	13	45
Total	65	298	30	54	78	448	40	125

Table S2. *Enterococcus faecium* and *Enterococcus faecalis* isolates collection cities in South Korea.

Bacterial species	No. of isolates (%)								Total
	Seoul	Gyeonggi	Busan	Daegu	Incheon	Gwangju	Daejeon	Ulsan	
E. faecium	95 (16.6)	33 (5.8)	180 (31.4)	19 (3.3)	38 (6.6)	21 (3.7)	56 (9.8)	131 (22.9)	573
E. faecalis	48 (13.6)	36 (10.2)	86 (24.4)	7 (2.0)	37 (10.5)	17 (4.8)	30 (8.5)	91 (25.9)	352
Total	143 (15.5)	69 (7.5)	266 (28.8)	26 (2.8)	75 (8.1)	38 (4.1)	86 (9.3)	222 (24.0)	925

Table S3. The MIC_{50} and MIC_{90} of the tested antimicrobials against *E. faecium* (n = 298) isolated from healthy dogs during 2020–2022 in South Korea.

Antimicrobials	2020 (n = 83)	2021 (n = 77)	2022 (n = 138)	Total (n = 298)
Ampicillin				
MIC_{50}	1	1	1	1
MIC_{90}	2	16	16	16
% Resistance (No. of isolates)	4.8 (4)	24.7 (19)	21.7 (30)	19.8 (71)
Chloramphenicol				
MIC_{50}	4	4	4	4
MIC_{90}	8	8	4	8
% Resistance (No. of isolates)	0 (0)	5.2 (4)	2.2 (3)	4.2 (15)
Ciprofloxacin				
MIC_{50}	2	4	2	2
MIC_{90}	8	16	8	16
% Resistance (No. of isolates)	42.2 (35)	55.8 (43)	32.6 (45)	44.3 (159)
Daptomycin				
MIC_{50}	4	4	4	4
MIC_{90}	4	4	4	4
% Resistance (No. of isolates)	2.4 (2)	3.9 (3)	0 (0)	2.2 (8)
Erythromycin				
MIC_{50}	4	4	4	4
MIC_{90}	8	64	64	64
% Resistance (No. of isolates)	28.9 (24)	46.8 (36)	43.5 (60)	42.6 (153)
Florfenicol				
MIC_{50}	2	4	2	2
MIC_{90}	4	4	2	4
% Resistance (No. of isolates)	0 (0)	1.3 (1)	2.2 (3)	2.2 (8)

Gentamicin				
MIC_{50}	128	128	128	128
MIC_{90}	128	2048	128	128
% Resistance (No. of isolates)	2.4 (2)	13 (10)	7.2 (10)	8.1 (29)
Kanamycin				
MIC_{50}	128	128	128	128
MIC_{90}	256	2048	2048	2048
% Resistance (No. of isolates)	7.2 (6)	18.2 (14)	11.6 (16)	15.3 (55)
Linezolid				
MIC_{50}	2	2	1	2
MIC_{90}	2	2	2	2
% Resistance (No. of isolates)	0 (0)	0 (0)	0 (0)	0 (0)
Quinupristin/dalfopristin				
MIC_{50}	2	2	2	2
MIC_{90}	2	4	2	2
% Resistance (No. of isolates)	2.4 (2)	15.6 (12)	5.8 (8)	8.6 (31)
Salinomycin				
MIC_{50}	2	2	2	2
MIC_{90}	2	2	2	2
% Resistance (No. of isolates)	0 (0)	0 (0)	0 (0)	0.6 (2)
Streptomycin				
MIC_{50}	128	128	128	128
MIC_{90}	128	2048	2048	2048
% Resistance (No. of isolates)	6 (5)	19.5 (15)	18.1 (25)	17 (61)
Tetracycline				
MIC_{50}	2	16	2	2
MIC_{90}	128	128	128	128
% Resistance (No. of isolates)	32.5 (27)	50.6 (39)	29 (40)	39.8 (143)
Tigecycline				
MIC_{50}	0.25	0.25	0.25	0.25
MIC_{90}	0.5	0.5	0.25	0.5
% Resistance (No. of isolates)	0 (0)	0 (0)	0 (0)	0 (0)
Tylosin				
MIC_{50}	4	4	2	4
MIC_{90}	8	64	64	64
% Resistance (No. of isolates)	7.2 (6)	24.7 (19)	13 (18)	17.5 (63)
Vancomycin				
MIC_{50}	2	2	2	2
MIC_{90}	2	2	2	2
% Resistance (No. of isolates)	0 (0)	0 (0)	0 (0)	0 (0)

MIC_{50} and MIC_{90} are the concentrations (μg/mL) at which 50% and 90% of the isolates were inhibited, respectively.

Table S4. The MIC_{50} and MIC_{90} of the tested antimicrobials against *E. faecium* (n = 54) isolated from healthy cats during 2020–2022 in South Korea.

Antimicrobials	2020 (n = 18)	2021 (n = 14)	2022 (n = 22)	Total (n = 54)
Ampicillin				
MIC_{50}	1	1	2	1
MIC_{90}	2	16	16	16

% Resistance (No. of isolates)	11.1 (2)	21.4 (3)	13.6 (3)	23.1 (18)
Chloramphenicol				
MIC_{50}	4	8	4	4
MIC_{90}	8	8	4	8
% Resistance (No. of isolates)	0 (0)	0 (0)	0 (0)	0 (0)
Ciprofloxacin				
MIC_{50}	1	4	2	2
MIC_{90}	8	16	8	16
% Resistance (No. of isolates)	33.3 (6)	71.4 (10)	36.4 (8)	48.7 (38)
Daptomycin				
MIC_{50}	4	4	4	4
MIC_{90}	4	4	4	4
% Resistance (No. of isolates)	11.1 (2)	0 (0)	0 (0)	5.1 (4)
Erythromycin				
MIC_{50}	4	4	8	4
MIC_{90}	64	64	64	64
% Resistance (No. of isolates)	16.7 (3)	28.6 (4)	54.5 (12)	34.6 (27)
Florfenicol				
MIC_{50}	2	4	2	2
MIC_{90}	4	4	2	4
% Resistance (No. of isolates)	0 (0)	0 (0)	0 (0)	0 (0)
Gentamicin				
MIC_{50}	128	128	128	128
MIC_{90}	128	2048	128	2048
% Resistance (No. of isolates)	5.6 (1)	14.3 (2)	9.1 (2)	14.1 (11)
Kanamycin				
MIC_{50}	128	128	128	128
MIC_{90}	2048	2048	256	2048
% Resistance (No. of isolates)	22.2 (4)	21.4 (3)	9.1 (2)	20.5 (16)
Linezolid				
MIC_{50}	2	2	2	2
MIC_{90}	2	2	2	2
% Resistance (No. of isolates)	0 (0)	0 (0)	0 (0)	0 (0)
Quinupristin/dalfopristin				
MIC_{50}	2	2	2	2
MIC_{90}	2	2	4	2
% Resistance (No. of isolates)	11.1 (2)	0 (0)	13.6 (3)	6.4 (5)
Salinomycin				
MIC_{50}	2	2	2	2
MIC_{90}	2	2	2	2
% Resistance (No. of isolates)	0 (0)	0 (0)	0 (0)	0 (0)
Streptomycin				
MIC_{50}	128	128	128	128
MIC_{90}	128	128	2048	2048
% Resistance (No. of isolates)	11.1 (2)	7.1 (1)	13.6 (3)	15.4 (12)
Tetracycline				
MIC_{50}	2	16	2	2
MIC_{90}	128	128	64	128
% Resistance (No. of isolates)	27.8 (5)	57.1 (8)	18.2 (4)	37.2 (29)
Tigecycline				

MIC_{50}	0.25	0.25	0.25	0.25
MIC_{90}	0.25	0.5	0.25	0.5
% Resistance (No. of isolates)	0 (0)	0 (0)	0 (0)	0 (0)
Tylosin				
MIC_{50}	4	4	4	4
MIC_{90}	64	64	64	64
% Resistance (No. of isolates)	16.7 (3)	14.3 (2)	13.6 (3)	19.2 (15)
Vancomycin				
MIC_{50}	2	2	2	2
MIC_{90}	2	2	2	2
% Resistance (No. of isolates)	0 (0)	0 (0)	0 (0)	0 (0)

MIC_{50} and MIC_{90} are the concentrations (μg/mL) at which 50% and 90% of the isolates were inhibited, respectively.

Table S5. The MIC_{50} and MIC_{90} of the tested antimicrobials against *E. faecalis* (n = 448) isolated from healthy dogs during 2020–2022 in South Korea.

Antimicrobials	2020 (n = 175)	2021 (n = 124)	2022 (n = 149)	Total (n = 448)
Ampicillin				
MIC_{50}	1	1	1	1
MIC_{90}	1	1	1	1
% Resistance (No. of isolates)	0 (0)	0 (0)	0 (0)	0 (0)
Chloramphenicol				
MIC_{50}	8	8	4	8
MIC_{90}	32	32	32	32
% Resistance (No. of isolates)	14.3 (25)	18.5 (23)	18.1 (27)	18.5 (104)
Ciprofloxacin				
MIC_{50}	1	1	1	1
MIC_{90}	2	2	1	2
% Resistance (No. of isolates)	3.4 (6)	4 (5)	2 (3)	3.4 (19)
Daptomycin				
MIC_{50}	1	1	1	1
MIC_{90}	2	2	2	2
% Resistance (No. of isolates)	0 (0)	0 (0)	0 (0)	0.4 (2)
Erythromycin				
MIC_{50}	2	2	2	2
MIC_{90}	64	64	64	64
% Resistance (No. of isolates)	40.6 (71)	37.9 (47)	39.6 (59)	42 (236)
Florfenicol				
MIC_{50}	2	2	2	2
MIC_{90}	4	4	2	4
% Resistance (No. of isolates)	3.4 (6)	4.8 (6)	1.3 (2)	2.7 (15)
Gentamicin				
MIC_{50}	128	128	128	128
MIC_{90}	2048	1024	2048	2048
% Resistance (No. of isolates)	18.9 (33)	12.9 (16)	20.8 (31)	19.2 (108)
Kanamycin				
MIC_{50}	128	128	128	128
MIC_{90}	2048	2048	2048	2048
% Resistance (No. of isolates)	31.4 (55)	31.5 (39)	31.5 (47)	33.5 (188)

Linezolid				
MIC_{50}	1	1	1	1
MIC_{90}	2	2	2	2
% Resistance (No. of isolates)	0 (0)	0 (0)	0 (0)	0 (0)
Quinupristin/dalfopristin				
MIC_{50}	8	8	8	8
MIC_{90}	8	8	8	8
% Resistance (No. of isolates)	ND	ND	ND	ND
Salinomycin				
MIC_{50}	2	2	2	2
MIC_{90}	2	2	2	2
% Resistance (No. of isolates)	0 (0)	0 (0)	0 (0)	0 (0)
Streptomycin				
MIC_{50}	128	128	128	128
MIC_{90}	2048	2048	2048	2048
% Resistance (No. of isolates)	19.4 (34)	18.5 (23)	18.1 (27)	20.5 (115)
Tetracycline				
MIC_{50}	32	64	32	64
MIC_{90}	128	128	128	128
% Resistance (No. of isolates)	62.9 (110)	67.7 (84)	65.8 (98)	67.3 (378)
Tigecycline				
MIC_{50}	0.25	0.25	0.25	0.25
MIC_{90}	0.5	0.5	0.5	0.5
% Resistance (No. of isolates)	0 (0)	0 (0)	0 (0)	0 (0)
Tylosin				
MIC_{50}	2	2	2	2
MIC_{90}	64	64	64	64
% Resistance (No. of isolates)	40.6 (71)	37.1 (46)	40.3 (60)	42 (236)
Vancomycin				hh
MIC_{50}	2	2	2	2
MIC_{90}	2	2	2	2
% Resistance (No. of isolates)	0 (0)	0 (0)	0 (0)	0(0)

MIC_{50} and MIC_{90} are the concentrations (μg/mL) at which 50% and 90% of the isolates were inhibited, respectively.

Table S6. The MIC_{50} and MIC_{90} of the tested antimicrobials against *E. faecalis* (n =125) isolated from healthy cats during 2020–2022 in South Korea.

Antimicrobials	2020 (n = 41)	2021 (n = 39)	2022 (n = 45)	Total (n = 125)
Ampicillin				
MIC_{50}	1	1	1	1
MIC_{90}	1	1	1	1
% Resistance (No. of isolates)	0 (0)	0 (0)	0 (0)	0 (0)
Chloramphenicol				
MIC_{50}	8	8	4	8
MIC_{90}	32	32	32	32
% Resistance (No. of isolates)	29.3 (12)	30.8 (12)	33.3 (15)	30.9 (46)
Ciprofloxacin				
MIC_{50}	1	1	1	1
MIC_{90}	2	2	2	2

% Resistance (No. of isolates)	7.3 (3)	5.1 (2)	4.4 (2)	6 (9)
Daptomycin				
MIC_{50}	2	1	1	2
MIC_{90}	2	2	2	2
% Resistance (No. of isolates)	0 (0)	0 (0)	0 (0)	0 (0)
Erythromycin				
MIC_{50}	2	4	64	4
MIC_{90}	64	64	64	64
% Resistance (No. of isolates)	46.3 (19)	46.2 (18)	55.6 (25)	47.7 (71)
Florfenicol				
MIC_{50}	2	2	2	2
MIC_{90}	4	4	16	4
% Resistance (No. of isolates)	4.9 (2)	7.7 (3)	11.1 (5)	7.4 (11)
Gentamicin				
MIC_{50}	128	128	128	128
MIC_{90}	2048	1024	2048	2048
% Resistance (No. of isolates)	26.8 (11)	20.5 (8)	35.6 (16)	28.2 (42)
Kanamycin				
MIC_{50}	128	128	128	128
MIC_{90}	2048	2048	2048	2048
% Resistance (No. of isolates)	39 (16)	28.2 (11)	40 (18)	36.2 (54)
Linezolid				
MIC_{50}	1	1	1	1
MIC_{90}	2	2	2	2
% Resistance (No. of isolates)	0 (0)	0 (0)	0 (0)	0 (0)
Quinupristin/dalfopristin				
MIC_{50}	8	8	8	8
MIC_{90}	8	16	16	8
% Resistance (No. of isolates)	ND	ND	ND	ND
Salinomycin				
MIC_{50}	2	2	2	2
MIC_{90}	2	2	2	2
% Resistance (No. of isolates)	0 (0)	0 (0)	0 (0)	0 (0)
Streptomycin				
MIC_{50}	128	128	128	128
MIC_{90}	2048	2048	2048	2048
% Resistance (No. of isolates)	19.5 (8)	17.9 (7)	33.3 (15)	24.8 (37)
Tetracycline				
MIC_{50}	64	64	64	64
MIC_{90}	64	64	128	128
% Resistance (No. of isolates)	80.5 (33)	76.9 (30)	68.9 (31)	73.8 (110)
Tigecycline				
MIC_{50}	0.25	0.25	0.25	0.25
MIC_{90}	0.25	0.25	0.5	0.5
% Resistance (No. of isolates)	0 (0)	0 (0)	0 (0)	0 (0)
Tylosin				
MIC_{50}	4	2	64	4
MIC_{90}	64	64	64	64
% Resistance (No. of isolates)	46.3 (19)	43.6 (17)	53.3 (24)	47 (70)
Vancomycin				
MIC_{50}	2	2	2	2

MIC_{90}	2	2	2	2
% Resistance (No. of isolates)	0 (0)	0 (0)	0 (0)	0 (0)
MDR	43.9 (18)	43.6 (17)	48.9 (22)	46.3 (69)

MIC_{50} and MIC_{90} are the concentrations (μg/mL) at which 50% and 90% of the isolates were inhibited, respectively.

Table S7. Antimicrobial resistance rate in *Enterococcus faecium* isolated from healthy dogs (n = 298) and cats (n = 54) during 2020–2022 in South Korea.

Antimicrobials	Resistance rate % (No. of isolates)									
	Dogs (n = 298)					Cats (n = 54)				
	2020 (n = 83)	2021 (n = 77)	2022 (n = 138)	Subtotal (n = 298)	*p*-value	2020 (n = 18)	2021 (n = 14)	2022 (n = 22)	Subtotal (n = 54)	*p*-value
Ampicillin	4.8 (4)*	24.7(19)	21.7 (30)	17.8 (53)	0.0037	11.1 (2)	21.4 (3)	13.6 (3)	14.8 (8)	0.4358
Chloramphenicol	0 (0)	5.2(4)	2.2 (3)	2.3 (7)	0.1272	0 (0)	0 (0)	0 (0)	0 (0)	ND
Ciprofloxacin	42.2 (35)	55.8(43)	32.6 (45)	41.3 (123)	0.8973	33.3 (6)	71.4 (10)	36.4 (8)	44.4 (24)	0.1073
Daptomycin	2.4 (2)	3.9(3)	0 (0)	1.7 (5)	0.7269	11.1 (2)*	0 (0)	0 (0)	3.7 (2)	0.0456
Erythromycin	28.9 (24)	46.8(36)	43.5 (60)	40.3 (120)	0.0902	16.7 (3)*	28.6 (4)	54.5 (12)*	35.2 (19)	0.0028
Florfenicol	0 (0)	1.3(1)	2.2 (3)	1.3 (4)	0.2527	0 (0)	0 (0)	0 (0)	0 (0)	ND
Gentamycin	2.4 (2)	13(10)	7.2 (10)	7.4 (22)	0.1015	5.6 (1)	14.3 (2)	9.1 (2)	9.3 (5)	0.3191
Kanamycin	7.2 (6)	18.2(14)	11.6 (16)	12. 1(36)	0.2406	22.2 (4)	21.4 (3)	9.1 (2)	16.7 (9)	0.3258
Linezolid	0 (0)	0(0)	0 (0)	0 (0)	ND	0 (0)	0 (0)	0 (0)	0 (0)	ND
Quinupristin/dalfopristin	2.4 (2)	15.6(12)	5.8 (8)	7.4 (22)	0.1015	11.1 (2)	0 (0)	13.6 (3)	9.3 (5)	0.6741
Salinomycin	0 (0)	0(0)	0 (0)	0 (0)	ND	0 (0)	0 (0)	0 (0)	0 (0)	ND
Streptomycin	6 (5)*	19.5(15)	18.1 (25)	15.1 (45)	0.0362	11.1 (2)	7.1 (1)	13.6 (3)	11.1 (6)	1
Tetracycline	32.5 (27)	50.6(39)	29 (40)	35.6 (106)	0.6437	27.8 (5)	57.1 (8)	18.2 (4)	31.5 (17)	0.5667
Tigecycline	0 (0)	0(0)	0 (0)	0 (0)	ND	0 (0)	0 (0)	0 (0)	0 (0)	ND
Tylosin tartrate	7.2 (6)	24.7(19)	13 (18)	14.4 (43)	0.1009	16.7 (3)	14.3 (2)	13.6 (3)	14.8 (8)	0.7123
Vancomycin	0 (0)	0 (0)	0 (0)	0 (0)	ND	0 (0)	0 (0)	0 (0)	0 (0)	ND

$p < 0.05$ was considered a significant change in antimicrobial resistance trend.

Table S8. Antimicrobial resistance rate in *Enterococcus faecalis* isolated from healthy dogs (n = 448) and cats (n = 125) during 2020–2022 in South Korea.

Antimicrobials	Resistance rate % (No. of isolates)									
	Dogs (n = 448)					Cats (n = 125)				
	2020 (n = 175)	2021 (n = 124)	2022 (n = 149)	Subtotal (n = 448)	*p*-value	2020 (n = 41)	2021 (n = 39)	2022 (n = 45)	Subtotal (n = 125)	*p*-value
Ampicillin	0 (0)	0 (0)	0 (0)	0 (0)	ND	0 (0)	0 (0)	0 (0)	0 (0)	ND
Chloramphenicol	14.3 (25)	18.5 (23)	18.1 (27)	16.7 (75)	0.6391	29.3 (12)	30.8 (12)	33.3 (15)	31.2 (39)	0.7699
Ciprofloxacin	3.4 (6)	4 (5)	2 (3)	3.1 (14)	0.9048	7.3 (3)	5.1 (2)	4.4 (2)	5.6 (7)	0.6246
Daptomycin	0 (0)	0 (0)	0 (0)	0 (0)	ND	0 (0)	0 (0)	0 (0)	0 (0)	ND
Erythromycin	40.6 (71)	37.9 (47)	39.6 (59)	39.5 (177)	0.8739	46.3 (19)	46.2 (18)	55.6 (25)	49.6 (62)	0.6404
Florfenicol	3.4 (6)	4.8 (6)	1.3 (2)	3.1 (14)	0.9048	4.9 (2)	7.7 (3)	11.1 (5)	8 (10)	0.3722
Gentamycin	18.9 (33)	12.9 (16)	20.8 (31)	17.9 (80)	0.8552	26.8 (11)	20.5 (8)	35.6 (16)	28 (35)	0.8491
Kanamycin	31.4 (55)	31.5 (39)	31.5 (47)	31.5 (141)	0.9878	39 (16)	28.2 (11)	40 (18)	36 (45)	0.6613
Linezolid	0 (0)	0 (0)	0 (0)	0 (0)	ND	0 (0)	0 (0)	0 (0)	0 (0)	ND
Quinupristin/dalfopristin	ND	ND	ND	ND	ND	ND	ND	ND	ND	ND
Salinomycin	0 (0)	0 (0)	0 (0)	0 (0)	ND	0 (0)	0 (0)	0 (0)	0 (0)	ND
Streptomycin	19.4 (34)	18.5 (23)	18.1 (27)	18.8 (84)	0.9141	19.5 (8)	17.9 (7)	33.3 (15)	24 (30)	0.4405
Tetracycline	62.9 (110)	67.7 (84)	65.8 (98)	65.2 (292)	0.7347	80.5 (33)	76.9 (30)	68.9 (31)	75.2 (94)	0.3668
Tigecycline	0 (0)	0 (0)	0 (0)	0 (0)	ND	0 (0)	0 (0)	0 (0)	0 (0)	ND
Tylosin tartrate	40.6 (71)	37.1 (46)	40.3 (60)	39.5 (177)	0.8739	46.3 (19)	43.6 (17)	53.3 (24)	48 (60)	0.8097
Vancomycin	0 (0)	0 (0)	0 (0)	0 (0)	ND	0 (0)	0 (0)	0 (0)	0 (0)	ND

$p < 0.05$ was considered a significant change in antimicrobial resistance trend. ND, non-defined.

Table S9. Antimicrobial resistance patterns of *Enterococccus faecium* isolated from healthy dogs (n = 298) during 2020–2022 in South Korea.

No. of antimicrobials (No. of isolates)	Resistance patterns (No. of isolates)
0 (n=68)	–
1 (n=114)	AMP (n=3), CIP (n=32), ERY (n=61), SYN (n=6), TET (n=12)
2 (n=54)	AMP CIP (n=2), AMP STR (n=4), AMP TET (n=1), CIP DAP (n=1), CIP ERY (n=6), CIP SYN (n=1), CIP TET (n=31), DAP ERY (n=1), DAP TET (n=1), ERY SYN (n=1), ERY TET (n=3), GEN KAN (n=1), STR TET (n=1)
3 (n=13)	AMP CIP STR (n=1), AMP CIP TET (n=3), AMP STR TET (n=3), CIP ERY TET (n=4), CIP KAN TET (n=1), ERY SYN TET (n=1)
4 (n=9)	AMP CHL FLR STR (n=1), AMP CIP KAN TET (n=1), AMP CIP STR TET (n=3), AMP ERY STR TYL (n=1), CIP ERY SYN TYL (n=1), CIP ERY TET TYL (n=1), GEN KAN STR TET (n=1)
5 (n=7)	AMP CIP ERY TET TYL (n=2), CHL CIP ERY TET TYL (n=2), CIP ERY STR TET TYL (n=2), ERY KAN STR TET TYL (n=1)
6 (n=8)	AMP CIP ERY KAN TET TYL (n=2), AMP CIP ERY STR TET TYL (n=2), CIP ERY KAN STR TET TYL (n=3), ERY KAN SYN STR TET TYL (n=1)
7 (n=10)	AMP CIP ERY GEN KAN TET TYL (n=2), AMP CIP ERY KAN STR TET TYL (n=4), AMP ERY GEN KAN STR TET TYL (n=3), CIP ERY KAN SYN STR TET TYL (n=1)
8 (n=5)	AMP CHL CIP ERY GEN KAN TET TYL (n=1), AMP CIP ERY FLR GEN KAN STR TYL (n=1), AMP CIP ERY GEN KAN SYN TET TYL (n=1), AMP CIP ERY GEN KAN STR TET TYL (n=2)
9 (n=7)	AMP CHL CIP ERY GEN KAN STR TET TYL (n=2), AMP CIP ERY FLR GEN KAN STR TET TYL (n=1), AMP CIP ERY GEN KAN SYN STR TET TYL (n=4)
10 (n=2)	AMP CIP DAP ERY GEN KAN SYN STR TET TYL (n=2)
11 (n=1)	AMP CHL CIP ERY FLR GEN KAN SYN STR TET TYL (n=1)

AMP, ampicillin; CIP, ciprofloxacin; CHL, chloramphenicol; DAP, daptomycin; ERY, erythromycin; FLR, florfenicol; GEN, gentamicin; KAN, kanamycin; SYN, quinupristin/dalfopristin; STR, streptomycin; TET, tetracycline; TYL, tylosin.

Table S10. Antimicrobial resistance patterns of *Enterococccus faecium* isolated from healthy cats (n = 54) during 2020–2022 in South Korea.

No. of antimicrobials (No. of isolates)	Resistance patterns (No. of isolates)
0 (n=15)	–
1 (n=19)	CIP (n=7), DAP (n=2), ERY (n=6), KAN (n=1), TET (n=3)
2 (n=7)	CIP ERY (n=2), CIP TET (n=4), ERY SYN (n=1)
3 (n=3)	AMP STR TET (n=1), CIP ERY SYN (n=1), CIP ERY TET (n=1)
4 (n=2)	AMP CIP ERY TYL (n=1), AMP CIP GEN KAN (n=1)
5 (n=2)	AMP CIP GEN KAN TET (n=1), CIP ERY KAN TET TYL (n=1)
6 (n=3)	AMP CIP ERY STR TET TYL (n=1), CIP ERY KAN STR TET TYL (n=1), ERY GEN KAN STR TET TYL (n=1)
7 (n=1)	AMP CIP ERY KAN SYN TET TYL (n=1)
8 (n=0)	–
9 (n=2)	AMP CIP ERY GEN KAN SYN STR TET TYL (n=2)
10 (n=0)	–

AMP, ampicillin; CIP, ciprofloxacin; DAP, daptomycin; ERY, erythromycin; GEN, gentamicin; KAN, kanamycin; SYN, quinupristin/dalfopristin; STR, streptomycin; TET, tetracycline; TYL, tylosin.

Table S11. Antimicrobial resistance patterns of *Enterococccus faecalis* isolated from healthy dogs (n = 448) during 2020–2022 in South Korea.

No. of antimicrobials (No of isolates)	Resistance patterns (No. of isolates)
0 (n=147)	–
1 (n=101)	KAN (n=1), TET (n=101)
2 (n=22)	CHL TET (n=11), CIP TET (n=3), ERY TET (n=1), ERY TYL (n=4), FLR TET (n=1), KAN TET (n=1), STR TET (n=1)
3 (n=25)	CHL ERY TYL (n=1), ERY KAN TET (n=1), ERY TET TYL (n=20), GEN KAN TET (n=1), KAN STR TET (n=2)
4 (n=31)	CHL ERY KAN TYL (n=1), CHL ERY TET TYL (n=12), CHL GEN KAN STR (n=1), CHL STR TET TYL (n=1), CIP ERY TET TYL (n=1), ERY FLR TET TYL (n=1), ERY KAN TET TYL (n=13), ERY STR TET TYL (n=1)
5 (n=56)	CHL ERY FLR TET TYL (n=1), CHL ERY KAN TET TYL (n=2), CHL ERY STR TET TYL (n=1), CHL KAN STR TET TYL (n=1), ERY GEN KAN TET TYL (n=24), ERY KAN STR TET TYL (n=27)
6 (n=40)	CHL ERY FLR KAN TET TYL (n=1), CHL ERY GEN KAN TET TYL (n=12), CHL ERY KAN STR TET TYL (n=5), CIP ERY FLR KAN TET TYL (n=1), CIP ERY GEN KAN TET TYL (n=1), CIP ERY KAN STR TET TYL (n=1), ERY FLR KAN STR TET TYL (n=1), ERY GEN KAN STR TET TYL (n=18)
7 (n=20)	CHL ERY FLR KAN STR TET TYL (n=3), CHL ERY GEN KAN STR TET TYL (n=16), CIP ERY GEN KAN STR TET TYL (n=1)
8 (n=4)	CHL CIP ERY FLR GEN KAN TET TYL (n=3), CHL CIP ERY GEN KAN STR TET TYL (n=1)
9 (n=2)	CHL CIP ERY FLR GEN KAN STR TET TYL (n=2)

CIP, ciprofloxacin; CHL, chloramphenicol; ERY, erythromycin; FLR, florfenicol; GEN, gentamicin; KAN, kanamycin; STR, streptomycin; TET, tetracycline; TYL, tylosin.

Table S12. Antimicrobial resistance patterns of *Enterococccus faecalis* isolated from healthy cats (n = 125) during 2020–2022 in South Korea.

No. of antimicrobials (No. of isolates)	Resistance patterns (No. of isolates)
0 (n=26)	–
1 (n=33)	CIP (n=1), ERY (n=1), TET (n=31)
2 (n=3)	CHL TET (n=1), CIP TET (n=1), ERY TET (n=1)
3 (n=8)	CHL FLR TET (n=1), CHL STR TET (n=1), ERY TET TYL (n=6)
4 (n=11)	CHL ERY TET TYL (n=8), CHL GEN KAN TET (n=1), ERY KAN TET TYL (n=2)
5 (n=10)	CHL ERY GEN KAN TYL (n=1), CHL ERY KAN TET TYL (n=1), ERY FLR STR TET TYL (n=1), ERY GEN KAN STR TYL (n=1), ERY GEN KAN TET TYL (n=3), ERY KAN STR TET TYL (n=3)
6 (n=21)	CHL CIP ERY FLR TET TYL (n=1), CHL ERY GEN KAN STR TYL (n=1), CHL ERY GEN KAN TET TYL (n=8), CHL ERY KAN STR TET TYL (n=2), CIP ERY KAN STR TET TYL (n=1), ERY GEN KAN STR TET TYL (n=8)
7 (n=6)	CHL ERY GEN KAN STR TET TYL (n=6)
8 (n=6)	CHL CIP ERY FLR GEN KAN TET TYL (n=1), CHL CIP ERY FLR KAN STR TET TYL (n=1), CHL ERY FLR GEN KAN STR TET TYL (n=4)
9 (n=1)	CHL CIP ERY FLR GEN KAN STR TET TYL (n=1)

CIP, ciprofloxacin; CHL, chloramphenicol; ERY, erythromycin; FLR, florfenicol; GEN, gentamicin; KAN, kanamycin; STR, streptomycin; TET, tetracycline; TYL, tylosin.
